# Academic Life in Emergency Medicine (ALiEM) Blog and Podcast Watch: Renal and Genitourinary Emergencies

**DOI:** 10.7759/cureus.3756

**Published:** 2018-12-20

**Authors:** Alice A Min, Jaime Jordan, Anand Swaminathan, Jacob Hennings, Andrew Grock

**Affiliations:** 1 Emergency Medicine, University of Arizona College of Medicine, Tucson, USA; 2 Emergency Medicine, University of California, Los Angeles, USA; 3 Emergency Medicine, New York University Bellevue, New York, USA; 4 Emergency Medicine, University of Tennessee Health Science Center College of Medicine, Chattanooga, USA

**Keywords:** emergency medicine, podcasts, blog posts, medical education

## Abstract

The Academic Life in Emergency Medicine (ALiEM) Approved Instructional Resources (AIR) series and Approved Instruction Resources Professional (AIR-Pro) series were created in 2014 and 2015, respectively, to address the need for the curation of online educational content as well as a nationally available curriculum that meets individualized interactive instruction criteria. These two programs identify high-quality educational blog and podcast content using an expert-based approach.

The AIR series is a continuously building curriculum originally based on the Council of Emergency Medicine Directors (CORD) testing schedule. Using the ALiEM AIR scoring instrument, 49 blog posts and podcasts relevant to renal and genitourinary emergencies published within the previous 12 months were evaluated by eight attending physicians.

We summarize the 13 posts that met our a priori determined quality criteria per evaluation by the reviewers.

The ALiEM Blog and Podcast Watch series identifies high-quality educational blogs and podcasts for emergency medicine clinicians through its expert panel, using a validated scoring instrument. While this article focuses on renal and genitourinary emergencies, additional AIR modules address other topics in emergency medicine. The AIR and AIR-Pro series provide post-publication accreditation and curation of recent online content to identify and recommend high-quality educational social media content for the EM clinician.

## Introduction and background

There has been a rapid rise in educational content available through blogs and podcasts in emergency medicine (EM) [1]. However, the identification of quality resources for educators and learners has only made preliminary progress [2-4]. In addition, in 2008, the Accreditation Council for Graduate Medical Education endorsed a decrease in synchronous conference experiences for EM residency programs by up to 20% in exchange for asynchronous learning - termed Individualized Interactive Instruction (III) [5]. These developments created a need for both an online content quality assessment as well as a nationalized curriculum that met III criteria.

To address these needs, the Academic Life in Emergency Medicine (ALiEM) Approved Instructional Resources (AIR) series and Approved Instructional Resources Professional (AIR-Pro) series were created in 2014 and 2015, respectively [6-7]. Using an expert-based, crowd-sourced approach, these two programs identify high-quality educational blog and podcast content. For the ALiEM Blog and Podcast Watch series, summaries of these posts are written by the AIR and AIR-Pro series’ editorial boards [8-10]. This installment from the series summarizes the highest scoring open-access educational resources on renal and genitourinary emergencies.

## Review

Topic identification

The AIR series is a continuously building curriculum originally based on the Council of Emergency Medicine Residency Directors (CORD) testing schedule.

Inclusion and exclusion criteria

A search of 50 of the most frequently visited sites per the Social Media Index [11-12] was conducted for resources relevant to renal and genitourinary emergencies, published within the previous 15 months. The search, conducted in May 2017, included blog posts and podcasts written in English for scoring by our expert panel.

Scoring

Extracted posts were scored without blinding by eight reviewers from the AIR Editorial Board, which is comprised of EM core faculty from various United States medical institutions. We utilized the ALiEM AIR scoring system [6-7], which is a validated scoring system that contains five measurement outcomes using seven-point Likert scales: Best Evidence in Emergency Medicine (BEEM) score, accuracy, educational utility, evidence-based, and references (Table 1) [13]. More detailed methods are described in the original description of the AIR series [6-7]. Reviewers with any role in the production of a reviewed resource recused him/herself from grading that resource.

**Table 1:** Approved Instructional Resources scoring instrument for blog and podcast content with the maximum score being 35 points. (BEEM = Best Evidence in Emergency Medicine; EP = emergency physician; EBM = Evidence-Based Medicine)

Tier 1: BEEM Rater Scale	Score	Tier 2: Content accuracy	Score	Tier 3: Educational Utility	Score	Tier 4: Evidence- Based Medicine	Score	Tier 5: Referenced	Score
Assuming that the results of this article are valid, how much does this article impact on EM clinical practice?		Do you have any concerns about the accuracy of the data presented or conclusions of this article?		Are there useful educational pearls in this article for senior residents?		Does this article reflect evidence-based medicine (EBM)?		Are the authors and literature clearly cited?	
Useless information	1	Yes, many concerns from many inaccuracies	1	Not required knowledge for a competent EP	1	Not EBM based, only expert opinion	1	No	1
Not really interesting, not really new, changes nothing	2		2		2		2		2
Interesting and new, but doesn't change practice	3	Yes, a major concern about few inaccuracies	3	Yes, but there are only a few (1-2) educational pearls that will make the EP a better practitioner to know or multiple (>=3) educational pearls that are interesting or potentially useful, but rarely required or helpful for the daily practice of an EP.	3	Minimally EBM based	3		3
Interesting and new, has the potential to change practice	4		4		4		4	Yes, authors and general references are listed (but no in-line references)	4
New and important: this would probably change practice for some EPs	5	Minimal concerns over minor inaccuracies	5	Yes, there are several (>=3) educational pearls that will make the EP a better practitioner to know, or a few (1-2) every competent EP must know in their practice	5	Mostly EBM based	5		5
New and important: this would change practice for most EPs	6		6		6		6		6
This is a "must know" for EPs	7	No concerns over inaccuracies	7	Yes, there are multiple educational pearls that every competent EP must know in their practice	7	Yes exclusively EBM based	7	Yes, authors and in-line references are provided	7

Resources with a mean score of greater than 30 points (out of a maximum of 35) are awarded the AIR label. Resources with a mean score of 27 to 28 and deemed accurate and educationally valuable by the reviewers are given the Honorable Mention (HM) label.

Results

We initially included a total of 49 blog posts and podcasts. Key educational pearls from the three AIR posts and the 10 Honorable Mentions are described.

*Article 1. Helman, A. BEEM Cases 2 - Renal Colic Imaging, Analgesia, Fluids & MET. (May 23, 2016) AIR *[14] *https://emergencymedicinecases.com/renal-colic/*

This article summarizes key issues in renal colic, specifically addressing optimal initial imaging, pain management, use of intravenous fluids, and medical expulsive therapy.

Take-home points: In the well-appearing patient with a presentation consistent with renal colic, no diagnostic imaging is necessarily indicated. Conversely, in the ill-appearing patient or in cases where there is suspicion of a clinically significant alternative diagnosis, a computed tomography (CT) scan should be performed. Imaging preference is less clear for patients not in either of these extremes. For these patients, either ultrasound or CT may be utilized, as there is insufficient evidence to favor one strategy or the other.

For analgesia in urolithiasis, studies evaluating nonsteroidal anti-inflammatory drugs (NSAIDs), acetaminophen, and morphine have been limited by inadequate doses of morphine. While NSAIDs are reasonable options for pain relief in urolithiasis, the authors recommend the early and appropriate use of intravenous (IV) opiates when necessary.

Limited evidence has not demonstrated a benefit of IV fluids for time to stone passage, need for surgical intervention, or pain relief. Thus, fluid should be administered at the discretion of their treating physician, taking into account their current fluid status.

Conflicting evidence regarding medical expulsion therapy for patients with renal colic exists. However, newer, more rigorous studies suggest that there is an absence of benefit for expulsive therapy in patients with small stones. There may be a benefit of expulsive therapy in the subset of patients with large (>5mm) stones.

*Article 2. Morgenstern, J. First10EM: UTI: More Than You Ever Wanted to Know. (April 15, 2017) AIR *[15] *https://first10em.com/uti/*

This article reviews the diagnosis and treatment of urinary tract infections (UTI).

Take-home points: Research is limited by the lack of a clear definition of a UTI. The author concludes that in healthy women, UTI can be diagnosed by history alone, especially with low clinical suspicion for sexually transmitted diseases or vaginitis. Urine cultures are not necessary for these patients. Additionally, many cases of uncomplicated cystitis will resolve on their own without antibiotics. If given, antibiotic selection should be based on local antibiograms and if symptoms persist for greater than 48 hours, patients should be reassessed. Chronically catheterized patients should also be treated on the basis of symptoms. Imaging is generally not necessary unless the patient is ill-appearing or in septic shock, there are concerns for obstruction, or in patients with pyelonephritis who have not improved after 48-72 hours of treatment. Asymptomatic bacteriuria should not be treated except in pregnant patients and those undergoing operative urologic procedures.

*Article 3. Swaminathan, A. CoreEM: Ureteral Colic. (July 6, 2016) AIR *[16] *https://coreem.net/core/ureteral-colic/*

This article summarizes the pathophysiology, presentation, diagnosis, and management of ureteral colic.

Take-home points: Ureteral colic is common and results in pain during the passage of stones (calcium oxalate, struvite, uric acid, cysteine) through the ureter, bladder, and urethra. Typical symptoms include a sudden onset of sharp flank pain with radiation to the abdomen/groin, commonly with associated nausea and vomiting. Hematuria is seen in 70%-90% of patients. The diagnosis can be made clinically in low-risk patients without a high suspicion for alternative diagnoses. While an ultrasound can be a reasonable initial diagnostic test, CT has the superior ability to identify alternative diagnoses. Treatment primarily focuses on pain control and the author recommends NSAIDs as the first line, with the adjunctive use of opioids. Here, they do not recommend routine medical expulsive therapy (MET), though it may be considered for large, distal stones.

*Article 4. Seupaul, T and Phan, M. SGEM#154: Here I Go Again, Kidney Stone. (May 12, 2016) HM *[17] *http://thesgem.com/2016/05/sgem154-here-i-go-again-kidney-stone/*

This blog post is a critical appraisal of two multicenter, randomized, placebo-controlled trials looking at medical expulsive therapy for ureteral colic treatment.

Take-home points: MET, primarily with tamsulosin, has been touted for over a decade as a useful adjunct in the management of ureteral colic. However, the original evidence for MET was low quality. In recent years, a number of well-done studies have shown an absence of benefit. The two studies reviewed in this podcast did not find any benefit to MET in all-comers with ureteral colic. A subgroup analysis in the second article by Furyk did find a number needed to treat (NNT) of 4.5 for stone passage at four weeks in patients with stones between 5 mm and 10 mm [18]. This finding is hypothesis-generating and will require further studies to see if a significant benefit is truly present. For now, the overwhelming evidence indicates a lack of benefit to MET for ureteral colic patients.

*Article 5. Sinert, R. EMDocs: Contrast-Induced Nephropathy: Confounding Causation. (February 3, 2017) HM *[19] *http://www.emdocs.net/contrast-induced-nephropathy-confounding-causation/*

This blog post discusses the history of contrast-induced nephropathy (CIN) and reviews recent evidence questioning the existence of this clinical entity.

Take-home points: CIN is defined as a 25% increase in serum creatinine (SCr) from the baseline or an absolute increase of 0.5 mg/dl 48-72 hours after exposure to intra-arterial or intravenous contrast media. The original studies looking at CIN found an association between the disease marker (SCr increase) and adverse outcomes. This linkage, though, was based on studies with a number of methodological flaws, including confounding factors and selection bias.

A recent article with an improved methodology failed to demonstrate a relationship between contrast exposure and the development of acute kidney injury (AKI). This study included patients with a baseline SCr of up to 4.0 mg/dl [20].

Based on this evidence, emergency providers should not fear CIN but rather should administer contrast, when necessary, to make a diagnosis.

*Article 6. Mallin, M; Dawson, M; Avila, J; Noble, V. Ultrasound Podcast: Renal Ultrasound. (April 27, 2016) HM *[21] *https://www.ultrasoundpodcast.com/2016/05/renal-ultrasound-continued-nobleultrasound-hydro-twinkling-jets-oh-foamed/*

This vodcast discusses the role of renal ultrasound in managing patients with suspected ureteral colic.

Take-home points: Imaging patients with symptoms consistent with ureteral colic is complicated and depends on a number of factors, including the history of stones, age, comorbid conditions, and risk assessment for other diagnoses. Obtaining a CT scan on all patients with ureteral colic leads to increased radiation exposure and costs without a clear patient benefit. Instead, the authors review algorithms directed at using point of care ultrasound (POCUS) for a number of patient groups and supplementing with low-dose CT scan and kidney-ureter-bladder (KUB) films, as necessary. Additionally, the group discusses the prognostic value of POCUS - if the patient has severe hydronephrosis, they are more likely to have a large stone and less likely to have spontaneous passage.

*Article 7. Schneider, A and Sanderson, W. EMDocs: Foley Catheter Patients: Common ED Presentations/Management/Pearls & Pitfalls (November 24, 2016) HM *[22] *http://www.emdocs.net/foley-catheter-patients-common-ed-presentations-management-pearls-pitfalls/*

This blog post discusses the evaluation, management, and potential complications of catheter-associated urinary tract infections (CA-UTI).

Take-home points: A CA-UTI diagnosis requires that the urinary catheter has been placed less than 48 hours previously, it was removed within the last 48 hours, or that the patient performs regular in-and-out catheterization. Important historical information to elicit includes the reason for catheter placement, who placed it, when it was placed, any complications with or since the placement, and the intended time interval for the placement. The physical examination is also important in order to discover any findings that may prompt a broader differential. Urinalysis results do not definitively diagnose UTI. New or changing symptoms are key for differentiating colonization from infection. Antibiotic selection should take previous cultures if available and the hospital’s antibiogram into consideration. If the catheter can be removed, change the catheter before the urinalysis and culture are sent. Consultation to urology should be made for all post-operative patients and possibly those with a history of difficult catheter placement before removing the catheter.

*Article 8. Mallin, M; Dawson, M; Avila, J; Noble, V. Ultrasound Podcast: Renal Ultrasound 2. (May 5, 2016) HM *[23] *http://www.ultrasoundpodcast.com/2016/05/renal-ultrasound-continued-nobleultrasound-hydro-twinkling-jets-oh-foamed/*

This post covers the initial evaluation of flank pain using the STONE score and incorporating ultrasound.

Take-home points: The STONE score helps determine the likelihood that flank pain is secondary to renal stone. An initial ultrasound may allow the patient to forego a CT scan and its concomitant radiation. There is a good interobserver agreement in diagnosing hydronephrosis on ultrasound, and degree of hydronephrosis correlates with stone size. If combined, a high degree of certainty for renal colic and a normal ultrasound indicates that the patient is unlikely to require urologic intervention. The presenters discuss their approach to evaluating patients with flank pain using CT, the STONE score, and ultrasound. Alternative modalities to detect kidney stones include ultrasound alone, ultrasound with abdominal plain film, and ultrasound with low-dose CT. Finally, always consider alternative diagnoses such as a renal mass, pyelonephritis, appendicitis, hernia, or abdominal aortic aneurysm or dissection.

*Article 9. Rezaie, S. RebelEM: Contrast Induced Nephropathy (CIN): Fact or Myth? (March 20, 2017) HM* [24] *http://rebelem.com/contrast-induced-nephropathy-cin-fact-myth/*

This blog post critically appraises a retrospective cohort analysis of >17,000 patients undergoing a CT scan to evaluate for the incidence of acute kidney injury (AKI) [20].

Take-home points: Contrast media use was not associated with the increased development of AKI, chronic kidney disease (CKD), dialysis, or renal transplant. Factors associated with the increased probability of AKI include increased age, preexisting congestive heart failure or chronic kidney disease, hypoalbuminemia, or the administration of nephrotoxic medications. Patients with decreased renal function were less likely to receive contrast and those who received contrast were given more intravenous fluids. Limitations to this study include its retrospective nature and lack of information about nephroprotective interventions performed after patients departed the emergency department.

*Article 10. Johnson, C. EMDocs: Doc I can’t Pee, What Could It Be? Evaluation and Management of Acute Urinary Retention in the Emergency Department. (March 1, 2017) HM *[25] *http://www.emdocs.net/doc-cant-pee-evaluation-management-acute-urinary-retention-emergency-department/*

This blog post reviews acute urinary retention (AUR) pathophysiology, concerning history, and physical exam findings and covers four cases to illustrate specific examples.

Take-home points: Causes of AUR include obstructive, inflammatory, infectious, neurologic, and pharmacologic-related causes. Avoid an anchoring bias with known benign prostatic hyperplasia (BPH) and treat with a Foley catheter, an alpha-reductase inhibitor, and urology follow-up in three days. In women, intrauterine sources are common, as well as compression from stool burden. Most neurologic cases stem from diabetic neuropathy as 75% of patients with neuropathy will develop cystopathy, requiring treatment with scheduled voiding, cholinergic receptor agonist, and intermittent self-catheterization. Antidepressants, over-the-counter cold medicines, and non-steroidal anti-inflammatory drugs (NSAIDS) can cause AUR. The offending agent must be discontinued and Foley catheter placement may be required until bladder function returns. There is no benefit to adding an alpha-reductase inhibitor. In patients with fever and AUR, prostatitis should be suspected. If at risk for a sexually transmitted infection, patients should receive targeted antibiotic coverage for gonorrhea and chlamydia. Finally, patients should be admitted if serum creatinine increases by 0.5 in a two-week period or a 20% increase in creatinine from the baseline in chronic kidney disease.

*Article 11. Fox, S. Epididymitis in Children. (August 24, 2016) HM *[26] *https://pedemmorsels.com/epididymitis-in-children/*

This blog post reviews acute epididymitis in children.

Take-home points: Acute epididymitis is a common cause of acute scrotal pain in children, with the majority in prepubertal and early adolescent boys. In young children, the incidence of bacterial infection is low, approximately 15% will have positive urine cultures. Despite this, the majority of patients receive antibiotics. The true etiology is unclear in most cases but may be due to inflammation from adjacent torsion of the appendix or the testis, reflux of sterile urine, viral illness, anatomic abnormalities, or trauma. Epididymitis is usually unilateral, with the right side more often affected. In the evaluation of scrotal pain, testicular torsion must be considered, with a low threshold for obtaining an ultrasound. If boys are not sexually active, they are likely low risk for an infectious etiology of epididymitis. Treatment based on abnormal urinalysis may be considered. Alternatively, holding treatment until urine culture results are obtained may be a reasonable option. In sexually active boys with an increased risk for sexually transmitted infections, consider testing and starting empiric therapy.

*Article 12. Farkas, J. PulmCrit Wee: Is Piperacillin-Tazobactam Nephrotoxic. (July 9, 2016) HM *[27] *https://emcrit.org/pulmcrit/piperacillin-tazobactam-nephrotoxic/*

This blog post discusses the nephrotoxicity associated with piperacillin-tazobactam.

Take-home points: A study by Jensen et al. demonstrated that patients on piperacillin-tazobactam experienced a rise in serum creatinine [28]. Animal studies, however, have demonstrated the possible nephroprotective effects of this drug by competitively inhibiting the organic anion transport system. This mechanism may also explain the rising serum creatinine by reducing creatinine secretion, presenting a “pseudo-nephrotoxicity” phenomenon. Recent studies have also shown that patients treated with piperacillin-tazobactam had higher rates of acute kidney injury but lower rates of dialysis, further supporting the idea that the rise in serum creatinine due to this medication may not be clinically relevant.

*Article 13. Horezco, T. PEM Playbook: Urine Trouble. (February 2, 2017) HM *[29] *http://pemplaybook.org/podcast/urine-trouble/*

This blog post discusses the evaluation and management of UTI in pediatric patients.

Take-home points: For the clinical symptoms of a UTI, including dysuria, urgency, frequency in a verbal child, or nondescript abdominal pain or vomiting in a well-appearing child, a urine specimen should be obtained. Before investigating for UTI, patients should be risk-stratified based on age, the presence of fever, and the absence of another source of infection. Bag urine samples should not be used; a catheterized specimen is always preferred. The definition of a UTI is pyuria and at least 50,000 colonies per mL of a single uropathogen. The presence of five or greater white blood cells per high power field is required. Nitrites are poorly sensitive in children as it takes four hours for nitrites to form and most children this age do not hold their urine. Simple UTIs should be treated for at least seven days and pyelonephritis for 10 to 14 days. Cephalexin, nitrofurantoin, and cefdinir are preferred antibiotics while sulfamethoxazole and trimethoprim are not favored due to increased resistance and the risk of Stevens-Johnson Syndrome. Any febrile UTI in infants younger than two months should be admitted and admission for children less than four to six months of age should be considered. Routine culture should be performed until approximately 10 years of age. Follow-up and imaging may be indicated to diagnose anatomic anomalies.

## Conclusions

The ALiEM Blog and Podcast Watch series' reviewers identified 13 high-quality educational blogs and podcasts for EM clinicians through its expert panel, using the ALiEM AIR scoring instrument. These social media resources are currently curated in the ALiEM AIR and AIR-Pro series, originally created to address EM residency needs. While this article focuses on renal and genitourinary emergencies, additional AIR modules address other topics in EM. The resources chosen specifically for renal/genitourinary diseases are herein shared and summarized to help clinicians and educators filter through the rapidly published multitude of blog posts and podcasts. Our search was limited to content produced within the previous 12 months from the top 50 Social Media Index sites. While these lists are by no means a comprehensive analysis of the entire Internet for this topic, the AIR and AIR-Pro series provide post-publication accreditation and curation of recent online content to identify and recommend high-quality educational social media content for the EM clinician.
